# Intimate partner violence and maternal depression during pregnancy: A community-based cross-sectional study in Ethiopia

**DOI:** 10.1371/journal.pone.0220003

**Published:** 2019-07-31

**Authors:** Sewhareg Belay, Ayalew Astatkie, Maria Emmelin, Sven Gudmund Hinderaker

**Affiliations:** 1 School of Public Health, College of Medicine and Health Sciences, Hawassa University, Hawassa, Ethiopia; 2 Department of Social Medicine and Global Health, Lund University, Lund, Sweden; 3 Centre for International Health, University of Bergen, Bergen, Norway; Stellenbosch University, SOUTH AFRICA

## Abstract

**Introduction:**

Intimate partner violence (IPV) is regarded an important public health and human rights issue, characterized by physical, sexual or emotional abuse. Globally more than one in three women report physical or sexual violence by their intimate partners. Though the association between IPV and depression is known, we found no study investigating depression as a risk factor for IPV and very few studies using standard tools in assessing both IPV and depression among pregnant women.

**Aim:**

To measure the prevalence of IPV and depression during pregnancy and assess the association between IPV and depression and other determinants.

**Methods:**

A community-based cross-sectional study was conducted among 589 pregnant women living in Wondo-Genet district, southern Ethiopia. IPV experience was assessed using a structured questionnaire of the World Health Organization (WHO), and maternal depression was measured by the Edinburgh Postnatal Depression Scale (EPDS). Descriptive statistics were computed and multivariable logistic regression was carried out to estimate risk and adjust for confounders.

**Results:**

The overall prevalence of IPV was 21% (95% confidence interval [CI] = 18.1–24.7). After adjusting for potential confounders, increased risk of IPV remained among rural women (adjusted odds ratio[AOR] = 2.09; 95%CI = 1.06–4.09), women who had parental exposure to IPV (AOR = 14.00; 95%CI = 6.43–30.48), women whose pregnancy was not desired (AOR = 9.64; 95%CI = 3.44–27.03), women whose husbands used alcohol (AOR = 17.08; 95%CI = 3.83–76.19), women with depression (AOR = 4.71; 95%CI = 1.37–16.18) and women with low social support (AOR = 13.93; 95%CI = 6.98–27.77). The prevalence of antenatal depressive symptom (with EPDS score above 13) was 6.8% (95% CI 6.2–11.3). Increased risk of depression was found among women who had been exposed to IPV (AOR = 17.60; 95%CI = 6.18–50.10) and whose husbands use alcohol (AOR = 3.31; 95%CI = 1.33–8.24).

**Conclusion:**

One in five pregnant women experienced IPV and it was strongly associated with depression. Screening for IPV and depression at antenatal visits with referral to relevant care and service is recommended.

## Introduction

Intimate partner violence (IPV) is regarded as an important public health and human rights issue, and is characterized by physical, sexual or emotional abuse. Usually the woman is the victim. Globally, more than one in three women report having experienced physical or sexual violence by their intimate partner. According to a report developed by the World Health Organization (WHO), the London School of Hygiene and Tropical Medicine and the South African Medical Research Council, in all regions of Sub-Saharan-Africa, the prevalence of IPV among ever partnered women is above the global average of 30.0% [1, 2]. In Ethiopia, the reported life time prevalence of domestic violence against women ranges from 20% to 78% [3]. Several studies indicated that women who had experienced IPV before pregnancy, continued to suffer during pregnancy [4, 5]. Among pregnant women reported IPV prevalence ranges from 2% to 57% [6], as shown in a systematic review of mainly African studies. Pun and colleagues through their large prospective cohort study which recruited 2,004 pregnant women seeking antenatal care reported a 20% IPV during pregnancy [7]. The prevalence of IPV during pregnancy has been reported to be 30% in Tanzania [8], 35% in Vietnam [9] and 44% in Egypt [10]. A few studies conducted in Ethiopia reported the prevalence during the current pregnancy to range from 23% to 36% [11–14], suggesting that the magnitude is not far from the prevalence among non-pregnant women.

Previous research has shown that IPV during pregnancy is associated with fatal and non-fatal ill health for both the mother and the new-born. Demelash etal through their hospital based case-control study conducted among 129 cases and 258 controls demonstrated that mothers who experienced any type of IPV during pregnancy were three times more likely to have a newborn with low birth weight [11]. Sanchez etal through a case-control study showed that, exposure to any IPV during pregnancy had a two-fold increased odds of spontaneous preterm birth [15]. The association between IPV and low birth weight and preterm birth were also evidenced by studies conducted by Ibrahim etal [10], Hassan etal [16] and Koen etal [17]. A large cross-sectional survey conducted among 1180 pregnant women attending antenatal care in Dares Salaam, Tanzania by Mahenge and colleagues revealed significantly higher odds of post traumatic stress disorder, anxiety and depressive symptoms in women who experienced physical and or sexual IPV during pregnancy [18]. Several studies reported evidence in support of these associations [19, 20]. Smoking, alcohol use and poor utilization of maternal health care are also associated with IPV [5, 20–23].

The Ethiopian Health Sector Transformation Plan (HSTP) did not address the issue of violence in spite of a strong political momentum for addressing violence against women in health and development agenda globally [24]. Recent studies conducted in Addis Ababa [25] and rural Ethiopia [26] linked IPV with depression; however, one focused on postnatal depression and the other assessed only the emotional aspect of IPV, making comparison difficult. Although it is known that unrecognized and untreated antenatal depression can persist as post-natal depression, few studies were conducted on assessing the prevalence of antenatal depression.

We found very few studies from Ethiopia using standard tools in assessing both IPV and depression among pregnant women. Therefore, we aimed to study IPV and depression among pregnant women. Our specific objectives were to: 1) measure the prevalence of IPV during pregnancy; 2) measure the prevalence of antenatal depression; 3) assess determinants of IPV; and 4) assess the determinants of antenatal depression. This study is part of a larger study on intimate partner violence in Sidama zone, southern Ethiopia.

## Methods

### Study design

This was a community based cross-sectional study conducted as part of a prospective cohort study from February to August, 2017.

### Study setting

The study was conducted in Wondo Genet district which is one of the 19 districts located in Sidama zone of the Southern Nations, Nationalities and Peoples Region. The district had an estimated total population of 153,283 based on the 2007 population and housing census. The total population of reproductive age women was 35,715 and the expected number of pregnancy was 5,304 [27]. It has 3 urban and 12 rural kebeles and 16 health posts and 5 health centers serving the population.

### Study population

Pregnant women living in Wondo-Genet district were the target population for this study.

The study population was pregnant women with gestational age 25–34 weeks enlisted by the Health Extension Workers (HEWs), living in the selected two urban and three rural kebeles. Those not currently living with an intimate partner were excluded.

### Sample size and sampling

Sample size was estimated in order to have sufficient sample size to estimate the prevalence of IPV with a 5% precision, and calculated based on a presumed prevalence of 32% [13] and design effect of 1.5 to compensate for non-random sampling. Adding 10% for non-response settled for a sample size of 606.

Two urban and three rural Kebeles were selected purposively based on ethnic diversity, population size and convenience for data collectors. The pregnant women were enrolled through home visits using lists available at Health Extension Workers (HEWs). The sample size was allocated to each kebele based on the current available list of pregnant women provided by the one-to-five network leaders and HEWs. Pregnant women who fulfilled the inclusion criteria were consecutively enrolled in to the study until the required sample size was obtained.

### Data collection and quality control

Data was collected using a structured questionnaire which composed of socio-demographic and obstetric characteristics, exposure to IPV and depression and social support received from different people. The questionnaire was translated into local languages (Sidaamu afoo) and Amharic and back to English by an expert on the local languages to ensure consistency. The translation and back translation of the EPDS was checked by a psychiatrist. A pilot study was conducted before commencing the actual data collection. Data was collected by face-to face interview based on the questionnaire, and performed by five female field assistants. They had been trained for one week in interviewing techniques, based on WHO ethical guidelines for studies about violence experiences [28]. The data collection was closely supervised by two health officers and the principal investigator.

### Variables

The main outcome variables were intimate partner violence and depression during current pregnancy. IPV was assessed using questions adapted from the WHO multi-country study on women’s health and domestic violence against women questionnaire [29]. IPV exposure in “the past 12 month” in the WHO study was changed to “during this pregnancy” in this study since our focus was assessing IPV during pregnancy Intimate partner violence was separated into three types of violence; 1) Physical violence (partner had slapped her with the palm of the hand; forced something to fall on her that could harm her; pushed her, hit her with fist or something else; kicked, dragged or beat her up; purposively choked or burnt her; or tried or actually used weapons); 2) Sexual violence (partner had physically forced her to have sexual intercourse; had sexual intercourse when she did not want to, because she was scared of what her partner might do; or had forced her to do something sexual that she found shameful); 3) Emotional violence (partner had insulted or made her to feel bad about herself; had belittled or humiliated her in front of other people; had done things purposely to frighten or intimidate her; and had tried to harm someone she cared about during the current pregnancy). Intimate partner violence during current pregnancy was coded “yes”, if the woman had experienced any of the three types of violence. IPV was also used as a covariate for analysing risk of maternal depression.

Maternal depression was measured by ten questions of the Edinburgh Postnatal Depression Scale (EPDS) [30] validated in previous studies conducted in Ethiopia [31, 32]. Each of the EPDS items has a score of 0–3, which allowed the total score to range from 0–30. To identify women with depressive symptoms we used a cut- off point of 13 and above [33–35]. Reliability test was performed using Cronbach’s alpha and was found to be 0.83, which indicated a high level of internal consistency of the items in the scale. Maternal depression was also used as a covariate for IPV as an outcome.

The covariates included in the analysis were age (years), own and partner’s education (no education, primary and secondary and above), occupation (housewife and others), residence (rural and urban), income (<1500, 1500–2999 and >3000 Ethiopian Birr), age at first marriage (years), duration in marriage (years), parity (number of alive children), desired pregnancy (desired, not desired, don’t know), history of violence between parents (yes, no, don’t know), own and partner’s use of alcohol, khat and cigarettes in the last 30 days (yes, no) and social support was measured by six items of the Maternity Social Support Scale [36]. Each item of the MSSS has a score of 1–5, which allowed the total score to range from 6–30 (low<18, medium 18–23 and high social support 24–30).

### Data analysis

Data was double entered by two data entry clerks using Epi-Data v.3.1 software (Odense, Denmark) and was analyzed using SPSS version 20 software. Means, frequency and percentages were computed. Bivariate and multivariable logistic regression analysis was carried out to assess determinants of IPV and determinants of depression, and adjust for potential confounders. The multivariable model was built by entering variables with associations p<0.25 using “Enter” method. Multicollinearity was checked using Collinearity diagnostics. Model goodness of fit was assessed using Hosmer and Lemeshow goodness-of -fit. Statistical significance was set at p-value ≤0.05 and odds ratios with 95% confidence interval (CI) were reported.

### Ethics considerations

Ethical approval was obtained from the Institutional Review Board (IRB) at the College of Medicine and Health Sciences, Hawassa University (Ref No: IRB/006/09) and regional ethical committee of Western Norway (Ref No: 2016/1908/REK vest). In the study area generally people do not like to sign, as they are skeptical to signing any official document; informed oral consent was approved by IRB and was acceptable to participants. It was obtained from each participant, and recorded by the interviewer. The study followed the ethical and safety guidelines recommended by the World Health Organization [28]. All the interviews were done with only the participant woman present. Information about available support was given to all women who participated in the study and those who wanted psychological support, were referred to Kela health center to get counseling, and a woman requesting legal support was referred to a relevant body, supported by the study project.

## Results

Table 1 shows the socio-demographic characteristics of the participants. A total of 589 pregnant women out of 606 invited were interviewed and enrolled, making a response rate of 97%. The mean age of the participants was 25 years, ranging from 16 to 45 years. Almost half (49.2%) of the participants had attended primary education, while one in five had no formal education. The majority (80.1%) of the participants were housewives. Almost half (48.8%) of their husbands had attended secondary education.

**Table 1:** Socio–demographic characteristics of pregnant women in Wondo Genet district, Ethiopia, 2017.

Characteristics	Number (N = 589)	Percent (%)
**Age**		
15–19	41	7.0
20–24	192	32.6
25–29	254	43.1
≥30	102	17.3
**Parity**		
Nulli parous	129	21.9
Primi parous	155	26.3
Multi parous	246	41.8
Grand multi para	59	10
**Education**		
No education	145	24.6
Primary	290	49.2
Secondary and above	154	26.1
**Occupation**		
Housewife	472	80.1
Others^a^	117	19.9
**Religion**		
Protestant	540	91.7
Others^b^	49	8.3
**Ethnicity**		
Sidama	404	68.6
Oromo	99	16.8
Others^c^	86	14.6
**Residence**		
Rural	304	51.6
Urban	285	48.4
**Monthly income (Ethiopian birr)**		
<1500	273	46.3
1500–2999	214	36.3
≥3000	102	17.3
**Educational status of husband**		
No education	113	19.2
Primary	188	32.0
Secondary and above	287	48.8

^a^Others occupation = Merchant, Government employee, and Daily laborer

^b^Others religion = Orthodox Christian, Muslim and Catholic Christian

^c^Others ethnicity = Wolayta, Amhara, Gurage, Hadiya and Selte

Emotional abuse had been experienced by 86 of the pregnant women (14.6%), sexual abuse by 56 (9.5%) and physical abuse by 54 of the women (9.2%). Many had been exposed to several of these types of IPV. Intimate partner violence of any kind had been experienced by 125 out of 589 pregnant women, making the overall prevalence 21.2%.

Table 2 shows that there was an association between each type of IPV and depression during pregnancy (p<0.001).

**Table 2:** Association between the different types of IPV and depression.

	All	Depression		P-value
		Depressed	Not depressed	
**Emotional IPV**				
No	503	14 (2.8)	489 (97.2)	<0.001
Yes	86	26 (30.2)	60 (69.8)	
**Physical IPV**				
No	535	18 (3.4)	517 (96.6)	<0.001
Yes	54	22 (40.7)	32 (59.3)	
**Sexual IPV**				
No	533	18 (3.4)	515 (96.6)	<0.001
Yes	56	22 (39.3)	34 (60.7)	

Table 3 shows determinants of IPV among the participants. The adjusted risk of IPV was higher among pregnant women who were rural residents, who had as a child witnessed IPV among their parents, in which their pregnancy was not desired, reporting alcohol use by their husband, had low social support and for pregnant women who had depressive symptoms.

**Table 3:** Determinants of intimate partner violence among pregnant women in Wondo Genet district, Ethiopia, 2017.

Characteristics	Total participants	Participants with IPV	Crude odds ratio (95%CI)	Adjusted odds ratio^1^ (95%CI)
All participants	589	125		
**Age**				
15–19	41 (7.0%)	7 (17.1%)	0.47 (0.19–1.18)	0.58 (0.09–3.81)
20–24	192 (32.6%)	43 (22.4%)	0.66 (0.39–1.14)	0.74 (0.21–2.59)
25–29	254 (43.1%)	44 (17.3%)	**0.48 (0.28–0.82)**	0.72 (0.25–2.03)
>30	102 (17.3)	31 (30.4%)	1	1
**Education**				
No education	145 (24.6%)	26 (17.9%)	0.69 (0.42–1.14)	0.55 (0.24–1.25)
Primary	290 (49.2%)	70 (24.1%)	1	1
Secondary and above	154 (26.1%)	29 (18.8%)	0.73 (0.45–1.19)	0.99 (0.42–2.29)
**Occupation**				
Housewife	472 (80.1%)	86 (18.2%)	1	1
Others	117 (19.9%)	39 (33.3%)	**2.24 (1.43–3.52)**	1.91 (0.92–3.97)
**Residence**				
Rural	304 (51.6%)	70 (23.0%)	1.25 (0.84–1.86)	**2.09 (1.06–4.09)**
Urban	285 (48.4%)	55 (19.5%)	1	1
**Income**				
<1500	273 (46.3%)	70 (25.6%)	**2.00 (1.09–3.69)**	0.61 (0.22–1.70)
1500–2999	214 (36.3%)	40 (18.7%)	1.33 (0.70–2.55)	0.99 (0.35–2.78)
>3000	102 (17.3%)	15 (14.7%)	1	1
**Age at first marriage**				
12–18	338 (57.4%)	65 (19.2%)	0.74 (0.50–1.11)	
19–25	239 (40.6%)	58 (24.3%)	1	
26–32	12 (2.0%)	2 (16.7%)	0.62 (0.13–2.93)	
**Duration in marriage**				
<5 years	254 (43.1%)	54 (21.3%)	0.71 (0.42–1.18)	0.62 (0.19–2.04)
6–10 years	223 (37.9%)	40 (17.9%)	0.57 (0.33–0.98)	0.70 (0.25–1.95)
>10 years	112 (19.0%)	31 (27.7%)	1	1
**Parity**				
0	129 (21.9%)	26 (20.2%)	0.94 (0.57–1.54)	
1–4	401 (68.1%)	85 (21.2%)	1	
>4	59 (10.0%)	14 (23.7%)	1.16 (0.61–2.21)	
Violence between parents*				
No	352 (59.8%)	33 (9.4%)	1	1
Don’t know	116 (19.7%)	22 (19.0%)	**2.26 (1.26–4.07)**	1.50 (0.61–3.66)
Yes	118 (20.0%)	70 (59.3%)	**14.10 (8.44–23.55)**	**14.00 (6.43–30.48)**
**Pregnancy desiredness**				
Not desired	45 (7.6%)	31 (68.9%)	**10.76 (5.50–21.02)**	**9.64 (3.44–27.03)**
Don’t know	11 (1.9%)	3 (27.3%)	1.82 (0.47–7.00)	0.59 (0.06–5.81)
Desired	533 (90.5%)	91 (17.1%)	1	1
Husband’s education*				
None	113 (19.2%)	27 (23.9%)	0.97 (0.56–1.67)	1.12 (0.44–2.83)
Primary	188 (32.0%)	46 (24.5%)	1	1
Secondary & above	287 (48.8%)	52 (18.1%)	0.68 (0.44–1.07)	1.12 (0.52–2.45)
**Alcohol use by husband**				
No	527 (89.5%)	82 (15.6%)	1	1
Yes	62 (10.5%)	43 (69.4)	**12.28 (6.81–22.14)**	**17.08 (3.83–76.19)**
**Khat use by husband**				
No	526 (89.3%)	93 (17.7%)	1	1
Yes	63 (10.7%)	32 (50.8%)	**4.81 (2.79–8.27)**	1.30 (0.33–5.11)
**Depressive symptom**^2^				
No	549 (93.2%)	91 (16.6%)	1	1
Yes	40 (6.8%)	34 (85.0%)	**28.52 (11.64–69.91)**	**4.71 (1.37–16.18)**
**Social support**				
Low	166 (28.2%)	97 (58.4%)	**19.83 (12.12–32.44)**	**13.93 (6.98–27.77)**
High	423 (71.8%)	28 (6.6%)	1	1

^1^Adjusted for age, educational status of the mothers and their husband, occupation, residence, income, duration in marriage,violence between parent’s, pregnancy desiredness, alcohol and khat use by husband, depressive symptom and social support.

^2^According to the Edinburgh Postnatal Depression Scale

CI: confidence interval

*: missing information from few participants.

The prevalence of antenatal depressive symptom among the participants (with EPDS score above 13) was 6.8% (95% CI 6.2−11.3).

Table 4 shows determinants of depression among participants; women exposed to IPV had a much higher risk of depression. Women whose husbands drank alcohol had 3 times higher risk of depression.

**Table 4:** Determinants of depression^1^ among pregnant women in Wondo Genet district, Ethiopia, 2017.

Characteristics	Total participants N (%)	Participants with depression N (%)	Crude odds ratio (95%CI)	Adjusted odds ratio^2^ (95%CI)
All	589 (100)	40 (6.8)		
**Age**				
15–24	233 (39.6)	16 (6.9)	1	1
25–34	332 (56.4)	22 (6.6)	0.96 (0.49–1.88)	1.68 (0.68–4.14)
35–45	24 (4.0)	2 (8.3)	1.23 (0.27–5.72)	2.05 (0.32–13.23)
**Residence**				
Rural	304 (51.6)	25 (8.2)	1	1
Urban	285 (48.4)	15 (5.3)	0.62 (0.32–1.20)	0.56 (0.23–1.34)
**Income**				
<1500	273 (46.3)	32 (25.6)	**6.64 (1.56–28.23)**	4.06 (0.82–19.98)
1500–2999	214 (36.3)	6 (18.7)	1.44 (0.29–7.27)	0.97 (0.16–5.83)
≥3000	102 (17.3)	2 (2.0)	1	1
**Pregnancy desiredness**				
Not desired	45 (7.6)	8 (17.8)	**3.63 (1.55–8.47)**	1.12 (0.39–3.18)
Don’t know	11 (1.9)	2 (18.2)	3.73 (0.77–18.01)	2.66 (0.31–23.10)
Desired	533 (90.5)	30 (5.6)	1	1
**Exposure to IPV**				
No	464 (78.8)	6 (1.3)	1	1
Yes	125 (21.2)	34 (27.2)	**28.52 (11.64–69.91)**	**17.60 (6.18–50.10)**
**Education**				
No education	145 (24.6)	6 (4.1)	0.51 (0.19–1.40)	0.48 (0.13–1.72)
Primary	290 (49.2)	22 (7.6)	0.97 (0.47–2.02)	0.73 (0.28–1.88)
Secondary and above	154 (26.1)	12 (7.8)	1	1
**Occupation**				
Housewife	472 (80.1)	25 (5.3)	1	1
Others	117 (19.9)	15 (12.8)	**2.63 (1.34–5.17)**	1.65 (0.71–3.3.82)
**Alcohol use by husband**				
No	527 (89.5)	23 (4.4)	1	1
Yes	62 (10.5)	17 (27.4)	**8.28 (4.12–16.62)**	**3.31 (1.33–8.24)**
**Parents violence**				
No	352 (59.8)	8 (2.3)	1	1
Don’t know	116 (19.7)	13 (11.2)	5.43 (2.19–13.45)	3.42 (1.14–10.26)
Yes	118 (20.0)	19 (16.1)	8.25 (3.51–19.42)	1.74 (0.61–4.97)

^1^Depression was assessed using the Edinburgh Postnatal Depression Scale.

^2^Adjusted for age, educational status, occupation, residence, income, violence between parents, pregnancy desiredness, alcohol use by partner and exposure to IPV.

## Discussion

In this community based study in southern Ethiopia we found that more than 20% of pregnant women suffer from intimate partner violence: emotional violence in 15% of participants, physical in 10% and sexual in 10%, many with a combination. Pregnancy seems not to protect against IPV. We also found that around 7% of them suffered from clinical symptoms of depression. There was a very strong association between IPV and depression.

Our prevalence figures of IPV are consistent with other studies from sub-Saharan Africa focusing on IPV during pregnancy showing prevalence of 25.8% and 23% in Ethiopia [11, 12], 27.7% in Uganda and [37], 28.7% in Nigeria [38]. All these papers used standard and validated tools, had fairly large sample size, are fairly recent, and has a reliable selection of similar participants in terms of setting and residence. Our prevalence figure was also within the range indicated in a systematic review of African studies [6].

There are also some studies about IPV in Africa that showed a higher prevalence of IPV than ours. A community based cross sectional study conducted in Abay Chomen district of Ethiopia showed 44.5% [39] and in Hulet Ejju district of Ethiopia reported 32.2% [13]. The difference in prevalence might be explained by cultural differences of the study areas. In addition, these studies were done in predominantly rural areas, whereas ours was conducted both in rural and urban kebeles. Our finding also confirmed that there was more reported violence in the rural areas. A study conducted in Kenya reported 37% prevalence of IPV, and this study was conducted among pregnant women seeking antenatal care in a district hospital [40]. A review indicated frequent use of health service by women who have experienced IPV [20]. As the Kenyan study was conducted among women visiting a hospital the likelihood of getting women who have experienced IPV is higher than in a community based study. The authors of the Kenyan study themselves acknowledged that the study was conducted in one hospital and consequently that it may not be representative and the reported prevalence was higher than many other studies. Cultural differences could also account for such differences. A study in Tanzania reported 30.3% prevalence of IPV among pregnant women recruited before 24 weeks of gestation [8]. The higher consumption of alcohol by the participants (11%) of the Tanzanian study, and the significant association between alcohol consumption and exposure to IPV might result in higher prevalence in this Tanzanian study than ours. The higher prevalence in the Tanzanian study might also be due to more assertive women so that they tend to disclose IPV as supported by the Tanzanian DHS that more than half of the women who experienced violence sought help from someone to stop the violence [41].

A community based predominantly rural study from Ethiopia reported a 5% IPV prevalence [26]. The lower prevalence could be due to using a different assessment tool. A study from Ghana showed a lower prevalence of IPV (5%), which was collected using tools not standardized for IPV. They assessed only physical violence and used data collected during a demographic and health survey; such a multipurpose study might result in under−reporting as the topic is so sensitive we think they may have missed several cases [42]. A Nigerian study among women in a tertiary hospital reported a prevalence of 2.3%, there may be selection bias with many women of relatively higher socioeconomic status not representative for the community, as well as reporting bias [5].

In agreement with previous studies [5, 8, 14, 43–45], this study revealed that rural residence, parental exposure to IPV, undesired pregnancy, low social support, depression and use of alcohol by husbands were determinants of IPV. The higher prevalence in rural areas may be related to various misconceptions held by the community that accepts violence. Though the exact mechanism how social support reduces IPV exposure not known, various studies [8, 43, 44] indicated the link between the two.

Our prevalence figure of depressive symptoms was close to figures reported by three studies conducted in Ethiopia ranging from 10.8% to 12% [26, 46, 47]. It is also comparable with a situational analysis result in five low and middle income countries including Ethiopia [48], a study conducted in Malawi [49] and Nigeria [50]. This could be due to the similarity in the setting and time at which the data was collected (antenatal vs postnatal). However several studies reported a higher prevalence figure ranging 23% to 34% [25, 51–55]. Possible reasons might be differences in time at which the data was collected and the screening tool they used.

In order to design prevention strategies based on the patterns found, it is useful to know which specific type of IPV had been associated with depression. Our analysis revealed that all types of IPV were highly associated with depression. This result was evidenced by a population based study conducted on 720 pregnant women in rural Bangladesh which reported an association between physical violence and antepartum depressive symptoms [56]. Varma and colleagues also reported higher depression symptoms in pregnant women with history of sexual abuse [57]. This result highlights the need for due considerations for all types of IPV.

Consistent with previous studies conducted in Ethiopia [26, 33], Malawi [49], Nepal [58] and Bangladesh [59] in this study, exposure to IPV during pregnancy and alcohol use by husband were determinants of antenatal depressive symptoms. Intimate partner violence is among the chronic stressful conditions that increases the risk of depression. Several studies indicated that stressful life events are among the factors significantly associated with depressive symptoms [60–62].

The close association between IPV and depression is not surprising. It is very common to see the conditions in the same individuals, but it is not possible to say which one is cause and which one is effect. A systematic review and meta-analysis of longitudinal studies indicated a double risk of incident depressive symptoms among women exposed to IPV and a double risk of incident IPV among depressed women [63]. Depression can make the daily life miserable so that the partner uses IPV, or IPV may be so devastating that the woman develops depression. With our study design we cannot conclude about causation.

This study had several strengths. A fairly large sample size with a good response rate should reflect the situation in the study area. The use of standardized validated tools ensured a fair reliability and validity of our findings. Also, the interview setting ensured confidentiality which is crucial in sensitive topics. Still, the estimated prevalence of IPV in our study should probably be regarded as minimum because of the sensitive topic that makes over−reporting unlikely. The study also had some limitations. The study did not consider the presence of other co−morbid mental health conditions that could contribute to depression, such as anxiety and stress, which could create confounding effects.

## Conclusions

In our study one in five pregnant women experienced domestic violence, confirming that pregnancy does not protect from IPV; and it was strongly associated with depression. There is a need for a change in mentality in the society about IPV; this may help survivors of IPV to know that this it is not “normal” but wrong and illegal. Screening for IPV at routine antenatal care can make it more open, but must be combined with an action plan with links to relevant services. There is a need to increase community awareness about the harmful effects of alcohol use by husband in order to reduce alcohol related IPV and depression. Future studies should focus on testing interventions to prevent and reduce IPV.

## Supporting information

S1 File Questionnaire used to conduct the study in Wondo Genet district, Ethiopia, 2017. (PDF)

S2 File Raw data used to construct Tables 1, 2, 3 and 4 in Wondo Genet district, Ethiopia, 2017. (SAV)
