# Supplementary material for: Intimate partner violence and maternal depression during pregnancy: A community-based cross-sectional study in Ethiopia
Source: PLoS One. 2019 Jul 31;14(7):e0220003. doi: 10.1371/journal.pone.0220003 (PMC6668805; doi:10.1371/journal.pone.0220003)
Supplement: S1 File — (PDF) [file pone.0220003.s001.pdf]

I: English version questionnaire

Read this information sheet and informed consent form to the participant.

Good morning/good afternoon.
My name is__________________

I am a data collector for the study conducted in Wondo Genet Woreda to assess the health and social welfare of pregnant women and their newborns. We are based at Hawassa University. Your household has been selected by chance to participate in a research study on women's health, so if you agree I will ask some questions about your health and your use of health services. Your participation in this study is completely voluntary.

When the study is finished your name will be replaced by a number to keep your participation anonymized. You do not have to answer any of the questions if you do not want to, and you may end this talk at any time you want to. If you refuse to participate in this study it has no consequences for you. However, your honest answers to these questions we hope will help us improve women's health and the services provided.

You will not get any monetary/material benefit for participation in this study. If you agree to participate in the study, there will be an interview at the start (at enrollment), and two follow up visits later in pregnancy, and a visit as soon as possible after birth to measure the baby's birth weights

Do you have any questions? If you have any questions to be clarified further you can talk to the principal investigator Sewhareg Belay via +251 916 874105.

The interview may take 40 minutes.

Do you agree to be interviewed and followed up? Agree [ ] Disagree [ ]

If Disagrees:
Thank the participant for her time and END the interview.

If Agrees:
It is very important that we should talk in private, with only you and me (excluding visitors, neighbors, husband, relatives and children above two years)

Is now agood time to talk? Yes____ No____
Is this a good place to hold the interview? Yes____ No____
Or would you suggest another place to hold the interview?

If the time and the place are not convenient, take an appointment.
Appointment Date___________ Place__________

If the time and the place are convenient, continue the interview

Some of the topics are sensitive in nature and difficult to discuss, but many women have found it useful to have the opportunity to talk. Memorizing painful events may cause distress. Those women who need psychological support will be referred to Kela health center and Hawassa University referral hospital.

Name of interviewer---------------------------Signature------------Date (Ethiopian) ----/----/----

Name of the Kebele____________ Respondent's code:____________

Part I- Socio-demographic and economic characteristics of respondent.

N<u>o</u>	Questions	Response
101	Where is your place of residence?	Rural-------------------------------1<br>Urban------------------------------2
102	Your age in completed years	______________Years
103	What is the highest education status you have attained	Not able to read and write---------1<br>Able to read and write-------------2<br>Grade 1-6---------------------------3<br>Grade 7-12--------------------------4<br>college and above------------------5
104	What is your ethnic group?	Sidama------------------------------1<br>Oromo-------------------------------2<br>Amhara------------------------------3<br>Wolayta ----------------------------4<br>Gurage ------------------------------5<br>Others (specify)--------------------6
105	What is your husband's ethnic group?	Sidama------------------------------1<br>Oromo-------------------------------2<br>Amhara------------------------------3<br>Wolayta ----------------------------4<br>Gurage ------------------------------5<br>Others (specify)--------------------6
106	What is your religion	Protestant ---------------------------1<br>Muslim------------------------------2<br>Orthodox----------------------------3<br>Catholic-----------------------------4<br>Traditional belief -------------------5<br>Others(specify)---------------------6
107	What is your husband's religion	Protestant ---------------------------1<br>Muslim------------------------------2<br>Orthodox----------------------------3

		Catholic-----------------------------4<br>Traditional belief -------------------5<br>Others(specify)----------------------6
108	What is your occupation?<br>**Do not read the responses to the respondents**	Housewife --------------------------1<br>Government employee-------------2<br>Merchant ---------------------------3<br>Daily laborer-----------------------4<br>Student------------------------------5<br>Others(specify)---------------------6
109	What is your current marital status?	Never married----------------------1<br>Married------------------------------2<br>Divorced----------------------------3<br>Widowed----------------------------4<br>Unmarried but in stable union----5<br>Separate-----------------------------6
110	Duration with in marriage	_______________years
111	How much is your estimated average monthly income?	______________Ethiopian birr
112	Age at first marriage	___________years
113	Number of children born alive	___ children in total<br>.______ children with another man
114	Is the current pregnancy desired by your partner	No------------------------------------1<br>Don't know-------------------------2<br>Yes-----------------------------------3
115	Was there ever violence between your parents?	No------------------------------------0<br>Yes-----------------------------------1
116	Partner's educational status	Not able to read and write---------1<br>Able to read and write-------------2<br>Grade 1-6---------------------------3<br>Grade 7-12--------------------------4<br>College and above------------------5
117	Partner's occupational status	Government employee-------------1<br>Farmer-------------------------------2<br>Merchant ---------------------------3<br>Daily laborer-----------------------4<br>Student------------------------------5<br>Others(specify)---------------------6
118	Did you drink alcohol in the last 30	No------------------------------------0

	days?	Yes-----------------------------------1
119	If YES, how many days did you drink alcohol in the last 30 days?	_________days
120	Did your partner drink alcohol in the last 30 days?	No------------------------------------0<br>Yes-----------------------------------1
121	If YES, how many days did he drink alcohol in the last 30 days?	_________days
122	Did you chew Khat in the last 30 days?	No------------------------------------0<br>Yes-----------------------------------1
123	If yes, then how many days did you chew Khat in the last 30 days	_________days
124	Did your partner chew Khat in the last 30 days?	No------------------------------------0<br>Yes-----------------------------------1
125	If yes, then how many days did he chew Khat in the last 30 days	
126	Did you smoke cigarettes /used tobacco of any kind in the last 30 days?	No------------------------------------0<br>Yes-----------------------------------1
127	If yes, then how many cigarettes did you smoked in the last 24 hours?	_________cigarettes
128	Did your partner smoke cigarettes /used tobacco of any kind in the last 30 days?	No------------------------------------0<br>Yes-----------------------------------1
129	If yes, then how many cigarettes did he smoke in the last 24 hours?	_________cigarettes
130	Last menstrual period(LMP)	On_________
131	Gestational age in completed weeks estimated based on LMP	_________weeks
132	If LMP not known, guessed month	_________months
133	ANC follow up	No------------------------------------0<br>Yes-----------------------------------1
134	Prior history of preterm	No------------------------------------0<br>Yes-----------------------------------1
135	Prior history of still birth	No------------------------------------0<br>Yes-----------------------------------1
136	Maternal Middle Upper Arm Circumference (MUAC)	_________in cm

Part II: Emotional abuse by an intimate partner

No	Questions	Responses
201_a	Were you insulted by your partner or made to feel bad about yourself during the current pregnancy?	Yes-----------1<br>No------------0
201_b	Did you experience this type of violence at any time in the year before this pregnancy by your current partner?	Yes-----------1<br>No------------0
202_a	Were you belittled by your partner or humiliated in front of other people during the current pregnancy?	Yes-----------1<br>No------------0
202_b	Did you experience this type of violence at any time in the year before this pregnancy by your current partner?	Yes-----------1<br>No------------0
203_a	Are things done by your partner to scare or intimidate you on purpose, e.g. by the way he looked at you, by yelling or smashing things during the current pregnancy?	Yes-----------1<br>No------------0
203_b	Did you experience this type of violence at any time in the year before this pregnancy by your current partner?	Yes-----------1<br>No------------0
204_a	Has your partner threatened to hurt someone you cared about during the current pregnancy?	Yes-----------1<br>No------------0
204_b	Did you experience this type of violence at any time in the year before this pregnancy by your current partner?	Yes-----------1<br>No------------0

Part III Physical violence by an intimate partner

No	Questions	Responses
301_a	Did your partner slap or had something thrown at you that could hurt you during the current pregnancy?	Yes-----------1<br>No------------0
301_b	Did you experience this type of violence at any time in the year before this pregnancy by your current	Yes-----------1<br>No------------0

	partner?	
302_a	Did your partner pushed or shoved you during the current pregnancy?	Yes------------1<br>No-------------0
302_b	Did you experience this type of violence at any time in the year before this pregnancy by your current partner?	Yes------------1<br>No-------------0
303_a	Did your partner hit with fist or something else that could hurt you during the current pregnancy?	Yes------------1<br>No-------------0
303_b	Did you experience this type of violence at any time in the year before this pregnancy by your current partner?	Yes------------1<br>No-------------0
304_a	Did your partner kick, drag or beat you during the current pregnancy?	Yes------------1<br>No-------------0
304_b	Did you experience this type of violence at any time in the year before this pregnancy by your current partner?	Yes------------1<br>No-------------0
305_a	Did your partner choke or burn you on purpose during the current pregnancy?	Yes------------1<br>No-------------0
305_b	Did you experience this type of violence at any time in the year before this pregnancy by your current partner?	Yes------------1<br>No-------------0
306_a	Did your partner threaten to use (or actually use) a gun, knife, spear or other weapon against you during the current pregnancy?	Yes------------1<br>No-------------0
306_b	Did you experience this type of violence at any time in the year before this pregnancy by your current partner?	Yes------------1<br>No-------------0
307	Were you ever punched or kicked in the abdomen during the current pregnancy?	Yes------------1<br>No-------------0

Part IV: Sexual violence by an intimate partner

No	Questions	Responses
401_a	Were you physically forced to have sexual intercourse with your partner when you did not want to during the current pregnancy?	Yes-----------1<br>No------------0
401_b	Did you experience this type of violence at any time in the year before this pregnancy by your current partner?	Yes-----------1<br>No------------0
402_a	Did you have sexual intercourse with your partner when you did not want to because you were afraid of what your partner might do during the current pregnancy?	Yes-----------1<br>No------------0
402_b	Did you experience this type of violence at any time in the year before this pregnancy by your current partner?	Yes-----------1<br>No------------0
403_a	Were you forced by your partner to do something sexual that you found degrading or humiliating during the current pregnancy?	Yes-----------1<br>No------------0
403_b	Did you experience this type of violence at any time in the year before this pregnancy by your current partner?	Yes-----------1<br>No------------0
404	**If the woman answered yes to any of the violence questions (part II or part III or Part IV) then ask**<br>Compared to before you were pregnant, did the violence get less, stay about the same, or get worse while you were pregnant?	Got less--------------1<br>Stayed about the same------------------2<br>Got worse------------3<br>I don't know---------4

Part V: Feelings of depression.

Tell us the way you have been feeling in the past seven days including today.

S.No	Questions	Responses	
501	In the past seven days, have you been able to laugh and see the funny side of things?	Yes	As much as I always could--0
			Not quite so much now---1
		No	Definitely not so much now---------------------------------------2
			Not (hardly) at all--3
502	In the past seven days, have you looked forward with enjoyment to things?	Yes	As much as I ever did---0
			Rather less than I used to---1
		No	Definitely less than I used to-------------------------------------2
			Hardly at all--3
503	In the past seven days, have you blamed yourself unnecessarily when things went wrong?	No	Not very often--0
			Never--1
		yes	Most of the time--2
			Some of the time--3
504	In the past seven days, have you been anxious or worried for no good reason?	No	Not at all--0
			Hardly ever---1
		Yes	Some times---2
			Very often--3
505	In the past seven days, have you felt scared or panicky for no very good reason?	No	Not at all---0
			Not much--1
		Yes	Some times---2
			Quite a lot---3

506	In the past seven days, have things been getting on top of you?	No<br><br>Yes	Coping as usual---0<br>Mostly able --1<br>Sometimes unable---2<br>Most of the time unable to cope --------------------------------3
507	In the past seven days, have you been so unhappy that you have had difficulty sleeping?	No<br><br>Yes	Not at all --0<br>Not very often ---1<br>Sometimes---2<br>Most of the time ---3
508	In the past seven days, have you felt sad or miserable?	No<br><br>Yes	Not at all---0<br>Not very often--1<br>Sometimes---2<br>Most of the time---3
509	In the past seven days, have you been so unhappy that you have been crying?	No<br><br>Yes	Never--0<br>Only occasionally---1<br>Quite often---2<br>Most of the time---3
510	In the past seven days, has the thought of harming yourself occurred to you?	No<br><br>Yes	Never---0<br>Hardly ever---1<br>Sometimes---2<br>Quite often---3

Part VI: Social support received from different people

Tell us about the support you received from different people

		Always	Most of the time	Some of the time	Rarely	Never
601	I have good friends who support me.	5	4	3	2	1
602	My family is always there for me.	5	4	3	2	1
603	My husband/partner helps me a lot.	5	4	3	2	1
604	There is conflict with my husband/partner.	1	2	3	4	5
605	I feel controlled by my husband/partner.	1	2	3	4	5
606	I feel loved by my husband/partner.	5	4	3	2	1

Thank you for your time and effort in responding to these questions

End

II: Sidamu-Afoo language version questionnaire
Tajenna qaali xa'mote sumuumme borro

Tenne Tajenna qaali xa'mote sumuumme borro beeqqanchote nabawi

Keerella galitta/hositta. Su'miya __________________. Wodo Gannatete Woradi giddo godowinni noo meentinna haaro ilamino qaaqquulle lainohunni assinanni xiinxallora taje gamba asseemmate. Hedeweelcho ati tenne xiinxallo giddo beeqqatta gede dooramootta.Konni daafira xa'mote sumuu yiittaro fayyimmakkina fayyimmate sonqansho horonsi'rakki lainohunni boode xa'mo xa'meemmohe.

Horontanni baxxino coyi hee'ranno ikki gede su'mikki bayiicho kiirotenni riqimbanni. Su'mikkinna teessokki horontanni konni woraqati aana diborreessantanno; ikkeennano, Alliitinohe ikki xa'muwa heedhuha ikkiro dawartooti. Tenne xiinxallo beeqa hogakkinnino iillannohe qarri dino. Ikkollana kayiinni ati aattanketi halaalaancho dawaro xiinxallonkenni noo qarra roorse afatenna amuwu aanna iillitanno gawajjo buxatanna maa assa hasiissannoro hedate qoleno woyaano coye kalaqatenna loosu aana hosiisate kaa'litanno. Ati aattanke taje tenne xiinxallo gobbaanni wole hajora horontanni dihosanno.

Beeqqaanchimmakkinni tenne xiinxallo giddonni afi'rattari womaashuno ikko uduunnichu irko horontanni dino. Xiinxallo beeqate summuu yiittaro hanafote xa'mo xa'meemohe. Saeno,illate gamba yaatta goofimarchu agannara lame marro hinge mine towanteemmohe qoleno illatta woyiite dandiinummo derrinni qaaqu ayiirre bikkate mulenni towanteemmohe.

Xa'mo afi'rootta? Xawinsannihe gede hasi'ratta xa'muwa nooheha ikkiro qara xiinxallancho bilbilte xa'ma dandaatta (Sewhareg Balay= +251 916 874105).

Qaali xa'mo 40 daqiiqi geeshsha adhitara dandiitanno.

Xa'mamate sumuu yaatta? Ee [ ] Dee'ni [ ]

sumuu yaa hoogguro galatte kae uurrisi.

Sumuu yituha ikkiro

Hojja hassawa lowo geesha hassisano . yaano atinna ani calla(ollu,minnikki anni, fiixikki woy oosso) nookiha yaate .

Xa hasaawate yanna injote? Ee [ ] Dee'ni [ ]

Bayiichu injannoke? Ee [ ] Dee'ni [ ]

Qaali xa'mote wolu injanno bayiichi no?

Yannanna bayiichu inja hoogiro dinye amaxxe. Dinyete barra___________ bayicho_________

Yannanna bayiichu injotero qaali xa'mo hanafi.

Mitte mitte xa'mo qorowo hasiissanootanna xa'mate ijjitanno ikkita ikkitara dandiitanno. Ikkollana kayiinni haammatu amuwi hasaawa dandaansa horote gede la'anno.sainota qarru yanna qaaga dadillissansara dandiitanno. Wodanu hiiqqamme Amaale hassiissanno amuwi hee'riro keellu fayyimmate mereershanna hawassa yuniveriste riferaale hosipiitaale sonkanni.

Sidaamu Afii Qaali-xa'muwa

Xa'maanchote Su'ma____________________ Malaate________________ Barra ____/____/____

Olluu Su'ma________________ Koode ________________

Biddissa I – Dawaraanchonniti dagoomittetenna miinju deerri akeeka.

**Kiiro**	**Xa'muwa**	**Dawaro**
101	Mama hee'ratta?	Baadiyyete------------------------------------1<br>Quchumaho-----------------------------------2
102	Gunde sa'ni diri giddo dirikki me"e ikkino?	_______________Diro
103	Roso ma deerra iillootta?	Nabbawanna borreessa didandeemma-----1<br>Nabbawanna borreessa dandeemma-------2<br>1-6 Kifile ------------------------------------3<br>7-12 Kifile -----------------------------------4<br>Kolleejjenna hakkunni alee-----------------5
104	Ga'rekki maati?	Sidaama---------------------------------------1<br>Oromo---2<br>Amaara---------------------------------------3<br>Wolayitta -------------------------------------4<br>Guraage --------------------------------------5<br>Wole (qummi assi)--------------------------6
105	Ayiddaannikki ga're maati?	Sidaama---------------------------------------1<br>Oromo---2<br>Amaara--3<br>Wolayitta -------------------------------------4<br>Guraage --------------------------------------5<br>Wole (qummi assi)--------------------------6
106	Amma'nokki maati?	Protestaante ----------------------------------1<br>Islaama---------------------------------------2<br>Ortodokise------------------------------------3<br>Kaatolike--------------------------------------4<br>Budu amma'no--------------------------------5<br>Wole (qummi assi)--------------------------6

107	Ayiddaannikki ama'no maati?	Protestaante -----------------------------------1<br>Islaama---2<br>Ortodokise-------------------------------------3<br>Kaatolike---------------------------------------4<br>Budu amma'no--------------------------------5<br>Wole (qummi assi)---------------------------6
108	Loosikki maati?<br>**Dawaraasinete dawaro nabbabbooti**	Minaama --------------------------------------1<br>Mangistete loosaasincho---------------------2<br>daddalaasincho -------------------------------3<br>Barru loosaasincho---------------------------4<br>Rosaancho-------------------------------------5<br>Wole (qummi assi)---------------------------6
109	Adhammekki deerri hiittooho?	Horontanni diassi'roomma------------------1<br>Assi'roomma----------------------------------2<br>Tidhamoomma--------------------------------3<br>Gunnittete--------------------------------------4<br>Diassi'roomma, ikkennano geado<br>mittimma nooe -------------------------------5<br>Baxxe hee'reemma---------------------------6
110	Adhammetenni keeshshootta yanna mageeshshite?	_______________diro
111	Mereerima aganu eokki mageeshshaati?	______________Itophiyu birra
112	Assi'ritta woyte dirikki me"e ikkanno	___________ diro
113	Lubbotenni ilantino qaaqquuli kiiro me"ete?	xaphoomunni _______________qaaqquulle<br>Wolu anniwiinni___________ qaaqquulle
114	Xa godowakki ayiddaannikki baxeenna godowoo?	Dibaxino---------------------------------------1<br>Diafoomma------------------------------------2<br>Ee--3
115	Amakkinna annikki mereero gibbo affe egennootta?	Horontanni------------------------------------0<br>Ee--1<br>Diafoomma------------------------------------2
116	Ayiddaannikki rosi deerri maricho lawanno?	Nabbawanna borreessa didandaanno------1<br>Nabbawanna borreessa dandaanno---------2<br>1-6 Kifile ------------------------------------3<br>7-12 Kifile -----------------------------------4<br>Kolleejjenna hakkunni alee-----------------5
117	Ayiddaannikki loosi deerri ma lawanno?	Mangistete looso------------------------------1<br>Gawarete --------------------------------------2<br>Daddalaasinchoho-----------------------------3

		Barru loosaasinchooti-----------------------4<br>Rosaanchoho---------------------------------5<br>Wole (qummi assi)--------------------------6
118	sai 30 barri giddo Dimbisannore agge egennotta?	Dee’ni---0<br>Ee--1
119	dawarose Ee ikkituro sai 30 barri giddo me’e barra ganyite agitta?	____________barra
120	sai 30 barri giddo Ayiddaannikki dimbisannore agino?	Dee’ni---0<br>Ee--1
121	dawarose “Ee” yaatuha ikkiro, sai 30 barri giddo mageeshshi barra ganye agino?	____________barra
122	sai 30 barri giddo caate itte egennotta?	Dee’ni---0<br>Ee--1
123	dawarose Ee ikkituro sai 30 barri giddo me’e barra ganyite ititta?	______________barra
124	sai 30 barri giddo ayidaannikki caate itte egennewo?	Dee’ni---0<br>Ee--1
125	dawarose Ee ikkituro sai 30 barri giddo ayyidaannikki caate me’e barra ganye itewo?	______________barra
126	sai 30 barri giddo sigaara /siqa wiliishite egennotta?	Dee’ni---0<br>Ee--1
127	dawarose Ee ikkituro sai 30 barri giddo me’e barra ganyite wiliishitta?	______________barra
128	sai 30 barri giddo ayiddaannikki sigaara /siqa wiliishe egennewo?	Dee’ni---0<br>Ee--1
129	dawarose Ee ikkituro sai 24 sa’aate giddo ayidaannikki sigara/siqa me’e sigaara williishi?	______________barra
130	goofimarchoho Meyaricho Laootta Barri (MLB) mamooteeti?	_______________nni
131	Godowu agani lamalatenni.	____________________ lamala

132	Meyaaricho laino yanna afa hoogguro qaaggano agana	_____________________agana
133	Ilate albiidi amaalenna ha'runso xaa godowira assi'ra hanafootta?	Dee'ni --0<br>Ee --1
134	Albi ilatta wpyte yanniweelo ilamino qaaqqi no?	Dinoe ---0<br>Ee --1
135	Godowi giddo baino qaaqqi dhagge afi'rootta?	Dee'ni---0<br>Ee--1
136	Amate Dasu Doyicho Bikki (ADDB)	_________ sentimetire (sm)

Biddissa II: Ayiddaannuwiinnii iillitinoti akatu hekko.

Kiiro	Xa'muwa	Dawaro
201a	Godowittahu kawa ayiddaannikki meessaneete giwatta gede woy hisatatta ikki gede xone egenninohe?	Ee -------------1<br>Dee'ni--------0
201b	konni godowi albaanni ayidaannikki sai dirira mamooteno ikkiro hattoo gawajjo iillishe egennewohe?	Ee-------------1<br>Dee'ni--------0
202a	Godowittahu kawa ayiddaannikki wolu manni albaanni shollishe woy mannu woroonni ikkatta asse egenninohe?	Ee-------------1<br>Dee'ni--------0
202b	konni godowi albaanni ayidaannikki sai dirira mamooteno ikkiro hattoo gawajjo iillishe egennewohe?	Ee-------------1<br>Dee'ni--------0
203a	Godowittahu kawa ayiddaannikki ate waajjishiishate yee assannori no? (Lawishshu gede: la"anno garinni, raaretenni woy mitoricho olanni)	Ee-------------1<br>Dee'ni--------0
203b	konni godowi albaanni ayidaannikki sai dirira mamooteno ikkiro hattoo gawajjo iillishe egennewohe?	Ee-------------1<br>Dee'ni--------0
204a	Godowittahu kawa ayiddaannikki ati lossi'ratta qaaqqo woy atewidoonni dayno manna gawajjate waajjishiishe egennino?	Ee-------------1<br>Dee'ni--------0

204b	konni godowi albaanni ayidaannikki sai dirira mamooteno ikkiro hattoo gawajjo iillishe egennewohe?	Ee-------------1<br>Dee'ni--------0

Biddissa III – Ayiddaannuwiinni iillitannoti ganate hekko. Woroonni uynoonniti baalanti xa'muwa xaa yannara noota godowate yanna illachishshanno.

Kiiro	Xa'muwa	Dawaro
301a	Godowittahu kawa ayiddaannikki qaawaade woy mitoricho ole gawajje egenninohe?	Ee-------------1<br>Dee'ni--------0
301b	konni godowi albaanni ayidaannikki sai dirira mamooteno ikkiro hattoo gawajjo iillishe egennewohe?	Ee-------------1<br>Dee'ni--------0
302a	Godowittahu kawa ayiddaannikki tuge egenninohe?	Ee-------------1<br>Dee'ni--------0
302b	konni godowi albaanni ayidaannikki sai dirira mamooteno ikkiro hattoo gawajjo iillishe egennewohe?	Ee-------------1<br>Dee'ni--------0
303a	Godowittahu kawa ayiddaannikki tuntummotenni woy gawajjahera dandaannorichinni gane egenninohe?	Ee-------------1<br>Dee'ni--------0
303b	konni godowi albaanni ayidaannikki sai dirira mamooteno ikkiro hattoo gawajjo iillishe egennewohe?	Ee-------------1<br>Dee'ni--------0
304a	Godowittahu kawa ayiddaannikki lekkatenni gane, goshooshe woy hekki asse geneggninohe?	Ee-------------1<br>Dee'ni--------0
304b	konni godowi albaanni ayidaannikki sai dirira mamooteno ikkiro hattoo gawajjo iillishe egennewohe?	Ee-------------1<br>Dee'ni--------0
305a	Godowittahu kawa ayiddaannikki mitirichi ledo cife woy giire egenninohe?	Ee-------------1<br>Dee'ni--------0
305b	konni godowi albaanni ayidaannikki sai dirira mamooteno ikkiro hattoo gawajjo iillishe egennewohe?	Ee-------------1<br>Dee'ni--------0
306a	Godowittahu kawa ayiddaannikki qawwetenni, billawunni woy olu udiinnichinni waajjishiishe egenninohe?	Ee-------------1<br>Dee'n---------0
306b	konni godowi albaanni ayidaannikki sai dirira mamooteno ikkiro hattoo gawajjo iillishe egennewohe?	Ee-------------1<br>Dee'ni--------0

307	konni godowi heedheenna ayidaannikki godowa hekki asse gane egenewohe?	Ee------------1<br>Dee’ni--------0

Biddissa IV: Ayiddaannuwiinni iillitinoti siimu xaadi hekko

**Kiiro**	**Xa’muwa**	**Dawaro**
401a	Godowittahu kawa ayiddaannikki hasi’rittakkinni siimu xaado assate wolqatenni gadache egenninohe?	Ee---------------1<br>Dee’ni-----------0
401b	konni godowi albaanni ayidaannikki sai dirira mamooteno ikkiro hattoo gawajjo iillishe egennewohe?	Ee---------------1<br>Dee’ni-----------0
402a	Godowittahu kawa ayiddaannikki ledo hasi’rittakkinni waajjitanninna mitorcho assannoe yaatenni yaaddanni siimu xaado assite egennootta?	Ee---------------1<br>Dee’ni-----------0
402b	konni godowi albaanni ayidaannikki sai dirira mamooteno ikkiro hattoo gawajjo iillishe egennewohe?	Ee---------------1<br>Dee’ni-----------0
403a	Godowittahu kawa ayiddaannikki siimu xaadi aana saalsiisannorenna ayirrinye ba”atta gede gadadisiise egenninohe?	Ee---------------1<br>Dee’ni-----------0
403b	konni godowi albaanni ayidaannikki sai dirira mamooteno ikkiro hattoo gawajjo iillishe egennewohe?	Ee---------------1<br>Dee’ni-----------0
404	ama aleeni noo xa’muwara (bidisha 1/bidisha2/bidisha3)mitte xa’mora nafa ee yite dawarturo aante noo xa’mo xa’mi. godowitta kawa iillitinohe gawajjo albi gawajo ledo heewisiinsiro ajjinonso albi gedeentilanso lexxitino?	ajjino________1<br>albi gedentila__2<br>lexxitino______3<br>diafoomma____4

Biddissa V: Dadillu macciishshamme

Sa'u lamalanni kayse techo barri geeshsha noohe macciishshamme kulinke.

Kiiro	Xa'muwa	Dawaro
501	Sa'u lamala giddo oso'lanna ososhiishshanno coyibba la"ate kaayyo afi'rootta? Lawaishshu gede ososhiishannorichi ososhiishannohe?	Baalanka woyte asseemma gede danduumma geeshsha----0<br>Xa nafa hakka geeshsha di"ikkino -------------------------1<br>Addintanni coyi'niro xa horontanni hattoori dino--------2<br>Co'ontanni (lowo geeshsha qarrunni) ------------------------3
502	Sa'u lamala giddo coyibba hagiirrunni la"atta? Lawishshu gede saakki godobbeenna qaltanno barra quqquxamatenni agaratta? Wole lawishsha la'nummoro dikko uurritanno barra quqquxamatenni agadhatta? Wole kore lawannore la"atta woyte tashshi yaannohe/hagiidhatta?	Danduumma geeshsha baalanka woyte asseemma-----------0<br>Konni albaanni assa hoogummarono mito mito woyte -----1<br>Addintanni konni albaanni asse egennoommakki garinni---2<br>Horontanni asse diegennoomma (lowo geeshsha qarrunni) 3
503	Sa'u lamala giddo baino coyra mullurrinni hunate macciishshamme macciishshantannohe? Lawishshu gede qaaqqikki xissamiro meessakki hunootta assite hedatta?	Ee, low yannara ---0<br>Ee, mito mito woyte ---1<br>Lowonta ganye diasseemma------------------------------------2<br>Dee'ni, horontanni--3
504	Sa'u lamala giddo mullurinni ka'e yaadatta?	Dee'ni, di-yaadoomma---0<br>Hili geeshsha nafa yaade diegennoomma---------------------1<br>Ee, mito mito woyte ---2<br>Ee, lowo geeshsha ganye -------------------------------------3

505	Sa'u lamala giddo mullurinni ka'e masatta?	Dee'ni, di-yaadoomma --0<br>Dee'ni, mase diyoomma --------------------------------------1<br>Ee, mito mito woyte ---2<br>Ee, addinta lowo geeshsha ----------------------------------3
506	Sa'u lamala giddo coy baalu wolqakki aleenni ikkinoha lawannohe?	Dee'ni, alba asseemmante gede fulaalla noomma ----------0<br>Dee'ni, lowo yannara aninni dancha gede guxxeemma ----1<br>Ee, mito mito woyte albinte gede meessi'ya guxxe fule diafeemma ---2<br>Ee, lowo yanna dhuki'ya aleenni ikkinoha lawannoenka..3
507	Sa'u lamala giddo ikkado goxano afi'ra hoogatenni hisanyo hoogootta?	Dee'ni, horontanni --0<br>Duucha woyte di"ikkino --------------------------------------1<br>Ee, mito mito woyte ---2<br>Ee, lowo yanna --3
508	Sa'u lamala giddo yaaddo macciishshantinohe?	Dee'ni, horontanni ---0<br>Lowonta diganyitino---1<br>Ee, mito mito woyte---2<br>Ee, lowo yanna---3
509	Sa'u lamalanni kayisse wi'latta geeshsha dadillootta?	Dee'ni, horontanni ---0<br>Sae sae calla --1<br>Ee, Lowo geshsha ganye--2<br>Ee, lowo yanna---3
510	Sa'u lamala giddo meessakki gawajjate hedo daggannohe?	Horontanni--0<br>Hedenni diegennoomma---------------------------------------1<br>Mito mito woyte---2

		Ee, lowo geeshsha ganye--------------------------------------3

BiddissaVI: Mannuwiinni afi'roommati dagoomitte kaa'lo

Babbaxxino manniwiinni afi'rootta kaa'lo kulinke

Kiiro	Xa'muwa	Duucha woyte	Lowo yanna	Mito mito woyte	Sae sae	Horontanni
601	Kaa'litannoere dancha jaalla afi'roomma.	5	4	3	2	1
602	Qarramuma woyte baala maate'ya ledo nooe.	5	4	3	2	1
603	Ayiddaanni'ya lowo geeshsha kaa'lannoe.	5	4	3	2	1
604	Ayiddaanni'ya ledo gibbo afi'roomma.	1	2	3	4	5
605	Ayiddaanni'ya hekki assinoeha lawannoe.	1	2	3	4	5
606	Ayiddaanni'ya baxannoeha lawannoe.	5	4	3	2	1

Yannakki uyte xa'muwa'ya dawarootta daafiranna irkokkira lowo geeshsha galateeffateemmahe

Gumulo

III: Amharic language version questionnaire

መረጃ መስጫና ፍቃደኝነት መጠየቂያ ቅፅ

ይህን የመረጃ መስጫና ፍቃደኝነት መጠየቂያ ቅፅ ለተሳታፊዋ ያንብቡላቸው፡፡

ከቡር ተሳታፊ

እንደምን አደሩ/ዋሉ

ስሜ----------------------------------ይባላል፡፡ ስለ ነፍሰጡር ሴቶች እና ስለሚወልዱአቸው ህፃናት ጤና እና ማሕበራዊ ደህንነት ለማወቅ በወንዶ ገነት ለሚካሄደው ጥናት መረጃ ሰብሳቢ ነኝ፡፡እንደ እድል ሆኖ እርስዎ እዚህ ጥናት ውስጥ እንዲሳተፉ ተመርጠዋል፡፡ስለዚህ ከተስማሙ ስለ ጤንነትዎ እና የጤና አገልግሎት አጠቃቀምዎ ጥቂት ጥያቄዎችን እጠይቅዎታለሁ፡፡እዚህ ጥናት ውስጥ የሚሳተፉት ግን ፍቃደኛ ሲሆኑ ብቻ ነው፡፡

አንዳችም ልዩ ነገር እንዳይኖር ጥናቱ ሲያበቃ ስምዎት በቁጥር ይተካል፡፡ስምዎት እዚህ ቅፅ ላይ አይፃፍም ፡፡ የማይመችዎትን የትኛውንም ጥያቄ ያለመመለስ ይችላሉ እንዲሁም ይሄንን ቃለ መጠይቅ በየትኛው ሰአት ማቆም ይችላሉ፡፡ ጥናት ውስጥ አልሳተፍም በማለትዎ የሚደርስብዎት ነገር የለም፡፡ ነገር ግን ለጥያቄዎቹ የሚሰጡን እውነተኛ መልስ ችግሩን የበለጠ እንድንረዳና ተመሳሳይ ችግር የሚደርስባቸውን ለመርዳት መፍትሄ ለማቀድ እና ለመተግበር ይረዳናል፡፡ የሚሰጡን መረጃ ከምርምር ስራ ዉጪ ለምንም ተግባር አይዉልም፡፡ሚስጥራዊነቱ ሙሉ በሙሉ የተጠበቀ ነዉ፡፡

ጥናት ውስጥ በመሳተፍዎ የሚያገኙት የገንዘብም ሆነ የቁስ ጥቅም የለም፡፡ጥናቱ ውስጥ ለመሳተፍ ከተስማሙ በመጀመሪያ ጥናቱ ውስጥ ሲካተቱ ቃለ-መጠይቅ ይደረግልዎታል፡፡ ከዚያም በእርግዝናዎ የመጨረሻ ወራት ሁለት የክትትል ጉብኝት እና ከወሊድ በኃላ የህፃኑን ክብደት ለመለካት በተቻለ መጠን በፍጥነት ጉብኝት ይኖረዎታል፡፡

ጥያቄ ይኖርዎታል? እንዲብራራልዎት የሚፈልጉት ጥያቄ ካለ ዋናዋን የጥናቱን ተመራማሪ ሰውሀረግ በላይን በዚህ የስልክ ቁጥር(+251 916 874 105) መጠየቅ ይችላሉ፡፡

ቃለ መጠይቁ 40 ደቂቃ ሊወስድ ይችላል፡፡

በዚህ ጥናት ውስጥ ለመሳተፍ ይስማማሉ? *እስማማለሁ( )* *አልስማማም( )*

አልስማማም ካሉ፡

ተሳታፊዋን ለጊዜያቸው አመስግነዉ ያቁሙ፡፡

እስማማለሁ ካሉ፡

ለብቻችን ማውራት በጣም አስፈላጊ ነው፡፡ ማለትም እኔና እርስዎ ብቻ (ጎረቤት፤ባለቤትዎት፤ ዘመድ ወይም ልጆች ሳይኖሩ)

አሁን ለመነጋገር ምቹ ጊዜ ነው? *አዎን( )* *አይደለም( )*

ቃለ-መጠይቁን ለማድረግ ቦታውስ ምቹ ነው? *አዎን( )* *አይደለም( )*

ቃለ-መጠይቁን ለማድረግ የሚጠቁሙን ሌላ ምቹ ቦታ ይኖራል?

ጊዜውና ቦታው ካልተመቸ ሌላ ቀጠሮ ይውሰዱ፡፡ *የቀጠሮ ቀን------------* *የቀጠሮ ቦታ------- -----*

ጊዜውና ቦታው ከተመቸ ቃለ-መጠይቁን ይቀጥሉ

አንዳንዶቹ ርዕሶች ጥንቃቄ የሚሹ እና ለመነጋገር የሚከብዱ ሊሆኑ ይችላሉ፡፡ ነገርግን ብዙዎቹ ሴቶች ማውራት መቻላቸውን እንደ ጥቅም ያዩታል፡፡ አስቸጋሪ ሁኔታዎችን ማስታወስ ሊያስከፋ ይችላል፡፡ የስነልቦና ድጋፍ የሚፈልጉ ሴቶች ወደ ኬላ ጤና አጠባበቅ ጣቢያ እና ሀዋሳ ዩኒቨርሲቲ ሪፈራል ሆስፒታል ይላካሉ፡፡

አማርኛ መጠይቅ

ቃለ መጠይቁን የሚጠይቅ ሰው ስም-- ፊርማ---------------ቀን-----/-----/---------

ቀበሌ---------------------------------- **ኮድ**---------------------------

ክፍል አንድ፡ ማህበራዊ እና አከባቢያዊ ጥያቄዎች

**ተ.ቁ**	**ጥያቄዎች**	**መልስ**
101	የመኖሪያ ቦታዎ የት ነው?	ገጠር --1<br>ከተማ--2
102	እድሜዎ ስንት ነው?	-------------------------አመት
103	የትምህርት ደረጃዎ ምንድን ነው?	ማንበብ እና መፃፍ የማትችል-----------------1<br>ማንበብ እና መፃፍ የምትችል----------------2<br>1-6 የተማረች---------------------------------3<br>7-12 የተማረች ------------------------------4<br>ኮሌጅ እና ከዛ በላይ-------------------------5
104	የምን ብሔር አባል ነዎት?	ሲዳማ--1<br>ኦሮሞ---2<br>አማራ--3<br>ወላይታ--------------------------------------4<br>ጉራጌ---5<br>ሌላ(ይጥቀሱ)--------------------------------6

105	ባለቤትዎ የምን ብሄር አባል ነው?	ሲዳማ--1<br>ኦሮሞ--2<br>አማራ--------------------------------------3<br>ወላይታ-------------------------------------4<br>ጉራጌ--5<br>ሌላ(ይጥቀሱ)--------------------------------6
106	የምን ሀይማኖት ተከታይ ነዎት?	ፕሮቴስታንት----------------------------------1<br>ሙስሊም------------------------------------2<br>ኦርቶዶክስ-----------------------------------3<br>ካቶሊክ---------------------------------------4<br>ባህላዊ እምነት-------------------------------5<br>ሌላ(ይጥቀሱ)--------------------------------6
107	ባለቤትዎ የምን ሀይማኖት ተከታይ ነው?	ፕሮቴስታንት----------------------------------1<br>ሙስሊም------------------------------------2<br>ኦርቶዶክስ-----------------------------------3<br>ካቶሊክ---------------------------------------4<br>ባህላዊ እምነት-------------------------------5<br>ሌላ(ይጥቀሱ)--------------------------------6
108	ስራዎ ምንድን ነው?<br>(ምርጫዎቹን አያንቡቡላቸው)	የቤት እመቤት--------------------------------1<br>የመንግስት ተቀጣሪ--------------------------2<br>ነጋዴ---3<br>የቀን ሰራተኛ----------------------------------4<br>ተማሪ--5<br>ሌላ(ይጥቀሱ)--------------------------------6

109	የጋብቻ ሁኔታዎ ምንድን ነው?	ያላገባች---------------------------------------1<br>ያገባች---2<br>የተፋታች--------------------------------------3<br>ባል የሞተባት--------------------------------4<br>በህግ ሳይጋቡ በአንድ ጣራ ስር የሚኖሩ-----5<br>በህግ ሳይለያዩ ተፋተው የሚኖሩ------------6
110	በጋብቻ ውስጥ ስንት አመታት ቆዩ?	______________አመታት
111	የወር ገቢዎ በግምት ምን ያህል ነው (በብር)?	______________ብር
112	የመጀመሪያ ባለቤትዎትን ሲያገቡ እድሜዎ ስንት ነበር?	______________አመት
113	በህይወት የተወለዱ ልጆች ብዛት	በጠቅላላ______________ልጆች<br>ከሌላ አባት______________ልጆች
114	ይህን እርግዝና ባለቤትዎት/አጋርዎት ይፈልጉታል?	አይደለም---------------------------------------1<br>አላውቅም---------------------------------------2<br>አዎን--3
115	በወላጆችዎ መካከል ጥቃት አጋጥሞ ያውቃል?	አይደለም---------------------------------------0<br>አዎን--1
116	የባለቤትዎ/አጋርዎ የትምህርት ደርጃ ምን ያህል ነው?	ማንበብ እና መፃፍ የሚችል-------------------1<br>ማንበብ እና መፃፍ የሚችል-------------------2<br>1-6 የተማረ-----------------------------------3<br>7-12 የተማረ----------------------------------4<br>ኮሌጅ እና ከዛ በላይ--------------------------5
117	የባለቤትዎ/አጋርዎ ስራ ምንድን ነው?	የመንግስት ተቀጣሪ----------------------------1<br>ገበሬ--2<br>ነጋዴ---3

		የቀን ሰራተኛ--------------------------------4<br>ተማሪ ---------------------------------------5<br>ሌላ(ይጥቀሱ)-------------------------------6
118	ባለፉት 30 ቀናት ውስጥ የአልኮል መጠጥ ጠጥተው ያውቃሉ ወይ?	አይደለም---------------------------------------0<br>አዎን--1
119	መልሳቸው አዎን ከሆነ ባለፉት 30 ቀናት ውስጥ ምን ያህል ቀናት ጠጡ?	______________ቀናት
120	ባለፉት 30 ቀናት ውስጥ ባለቤትዎት የአልኮል መጠጥ ጠጥተው ያውቃሉ ወይ?	አይደለም---------------------------------------0<br>አዎን--1
121	መልሳቸው አዎን ከሆነ ባለፉት 30 ቀናት ውስጥ ባለቤትዎት ምን ያህል ቀናት ጠጡ?	______________ቀናት
122	ባለፉት 30 ቀናት ውስጥ ጫት ቅመው ያውቃሉ ወይ?	አይደለም---------------------------------------0<br>አዎን--1
123	መልሳቸው አዎን ከሆነ ባለፉት 30 ቀናት ውስጥ ምን ያህል ቀናት ጫት ቃሙ?	______________ቀናት
124	ባለፉት 30 ቀናት ውስጥ ባለቤትዎት ጫት ቅመው ያውቃሉ ወይ?	አይደለም---------------------------------------0<br>አዎን--1
125	መልሳቸው አዎን ከሆነ ባለፉት 30 ቀናት ውስጥ ባለቤትዎት ምን ያህል ቀናት ጫት ቃሙ?	______________ቀናት
126	ባለፉት 30 ቀናት ውስጥ ሲጋራ/እንደ ትንባሆ የሚጨስ ነገር አጪሰው ያውቃሉ ወይ?	አይደለም---------------------------------------0<br>አዎን--1
127	መልሳቸው አዎን ከሆነ ባለፉት 24 ሰአት ውስጥ ስንት ሲጋራ አጨሱ?	______________ሲጋራ
128	ባለፉት 30 ቀናት ውስጥ ባለቤትዎት ሲጋራ/እንደ ትንባሆ የሚጨስ ነገር አጪሰው ያውቃሉ ወይ?	አይደለም---------------------------------------0<br>አዎን--1
129	መልሳቸው አዎን ከሆነ ባለቤትዎት ባለፉት 24 ሰአት ውስጥ ስንት ሲጋራ አጨሱ?	______________ሲጋራ

130	ለመጨረሻ ጊዜ ያዩት የወር አበባ መፍሰስ የጀመረበት ቀን	በ____________
131	የእርግዝናው እድሜ በሳምንት	____________ሳምንታት
132	በግምት የእርግዝናው እድሜ በወራት	____________ወራት
133	የነፍሰ ጡር ክትትል ማድረግ ጀምረዋል?	አይደለም---------------------------------------0<br>አዎን---1
134	ከዚህ በፊት ያለ ጊዜው የተወለደ ልጅ ነበረዎት?	አይደለም---------------------------------------0<br>አዎን---1
135	ከዚህ በፊት ሞቶ የተወለደ ልጅ ነበረዎት?	አይደለም---------------------------------------0<br>አዎን---1
136	የእናቲቱ የላይኛው ክንድ ዙሪያ በሳንቲ ሜትር ሲለካ	____________ሳንቲ ሜትር

ክፍል ሁለት፡ በባለቤት/በአጋር የሚደርስ ስሜታዊ ጥቃት

ተ.ቁ	ጥያቄዎች	መልስ
201ሀ	በዚህ እርግዝና ወቅት በባለቤትዎ ተሰድበው፤ ተዘልፈው ወይም ስለራስዎ መጥፎ ስሜት እንዲሰማዎ ተደርገው ያውቃሉ ወይ?	አዎን------------1<br>አይደለም----------0
201ለ	ከዚህ እርግዝና በፊት በባለፈው አመት በማንኛውም ጊዜ በአሁኑ ባለቤትዎት እንደዚህ አይነት ጥቃት አጋጥሞዎት ያውቃል ወይ?	አዎን------------1<br>አይደለም----------0
202ሀ	በዚህ እርግዝና ወቅት ባለቤትዎ በሌሎች ሰዎች ፊት አንሰው እንዲታዩ ወይም እንዲዋረዱ አደርገዎት ያውቃሉ ወይ?	አዎን------------1<br>አይደለም----------0
202ለ	ከዚህ እርግዝና በፊት በባለፈው አመት በማንኛውም ጊዜ በአሁኑ ባለቤትዎት እንደዚህ አይነት ጥቃት አጋጥሞዎት ያውቃል ወይ?	አዎን------------1<br>አይደለም----------0

203ሀ	በዚህ እርግዝና ወቅት ባለቤትዎ ሆን ብሎ እርስዎን ለማስፈራራት ወይም ለማስደንገጥ ነገሮችን ያደርጋሉ ወይ? ለምሳሌ በአስተያየት፤	አዎን------------1<br>አይደለም----------0
203ለ	ከዚህ እርግዝና በፊት በባለፈው አመት በማንኛውም ጊዜ በአሁኑ ባለቤትዎት እንደዚህ አይነት ጥቃት አጋጥሞዎት ያውቃል ወይ?	አዎን------------1<br>አይደለም----------0
204ሀ	በዚህ እርግዝና ወቅት ባለቤትዎ እርስዎ የሚወዱትን ሰው ለመጉዳት ተፈታትኖዎት ያውቃሉ ወይ?	አዎን------------1<br>አይደለም----------0
204ለ	ከዚህ እርግዝና በፊት በባለፈው አመት በማንኛውም ጊዜ በአሁኑ ባለቤትዎት እንደዚህ አይነት ጥቃት አጋጥሞዎት ያውቃል ወይ?	አዎን------------1<br>አይደለም----------0

ክፍል ሶስት፡ በባለቤት/በአጋር የሚደርስ አካላዊ ጥቃት፡፡ ከዚህ በታች የቀረቡት ጥያቄዎች በሙሉ የሚያመለክቱት ስለ አሁኑ እርግዝና ብቻ ነው፡፡

ተ.ቁ	ጥያቄዎች	መልስ
301ሀ	ባለቤትዎ በጥፊ መቶዎት ወይም ሊጎዳዎት የሚችል ነገር በዚህ እርግዝና ወቅት ወርውሮብዎት ያውቃሉ ወይ?	አዎን------------1<br>አይደለም--------0
301ለ	ከዚህ እርግዝና በፊት በባለፈው አመት በማንኛውም ጊዜ በአሁኑ ባለቤትዎት እንደዚህ አይነት ጥቃት አጋጥሞዎት ያውቃል ወይ?	አዎን------------1<br>አይደለም--------0
302ሀ	በዚህ እርግዝና ወቅት ባለቤትዎ ገፍቶዎት ወይም በሀይል ገፍቶዎት ያውቃሉ ወይ?	አዎን------------1<br>አይደለም--------0
302ለ	ከዚህ እርግዝና በፊት በባለፈው አመት በማንኛውም ጊዜ በአሁኑ ባለቤትዎት እንደዚህ አይነት ጥቃት አጋጥሞዎት ያውቃል ወይ?	አዎን------------1<br>አይደለም--------0
303ሀ	በዚህ እርግዝና ወቅት ባለቤትዎ በቡጢ ወይም በሌላ ሊጎዳዎ በሚችል ነገር መቶዎት ያውቃሉ ወይ?	አዎን------------1<br>አይደለም--------0
303ለ	ከዚህ እርግዝና በፊት በባለፈው አመት በማንኛውም ጊዜ በአሁኑ ባለቤትዎት እንደዚህ አይነት ጥቃት አጋጥሞዎት ያውቃል ወይ?	አዎን------------1<br>አይደለም----------0
304ሀ	በዚህ እርግዝና ወቅት ባለቤትዎ ረግጠዎት፤ ጎትቶዎት ወይም መቶዎት ያውቃሉ ወይ?	አዎን------------1<br>አይደለም-------0

304ለ	ከዚህ እርግዝና በፊት በባለፈው አመት በማንኛውም ጊዜ በአሁኑ ባለቤትዎት እንደዚህ አይነት ጥቃት አጋጥሞዎት ያውቃል ወይ?	አዎን-----------1<br>አይደለም---------0
305ሀ	በዚህ እርግዝና ወቅት ባለቤትዎ ሆን ብሎ አንቆዎት ወይም አቃጥሎዎት ያውቃሉ ወይ?	አዎን-----------1<br>አይደለም-------0
305ለ	ከዚህ እርግዝና በፊት በባለፈው አመት በማንኛውም ጊዜ በአሁኑ ባለቤትዎት እንደዚህ አይነት ጥቃት አጋጥሞዎት ያውቃል ወይ?	አዎን-----------1<br>አይደለም---------0
306ሀ	በዚህ እርግዝና ወቅት ባለቤትዎ በመሳሪያ ወይም በቢለዋ ሊጎዳዎት ሞክሮ ወይም ጎድቶዎት ያውቃሉ ወይ?	አዎን-----------1<br>አይደለም-------0
306ለ	ከዚህ እርግዝና በፊት በባለፈው አመት በማንኛውም ጊዜ በአሁኑ ባለቤትዎት እንደዚህ አይነት ጥቃት አጋጥሞዎት ያውቃል ወይ?	አዎን-----------1<br>አይደለም---------0
307	በዚህ እርግዝና ወቅት ሆድሽ ላይ በጡጫ ተመተሽ ታውቂያለሽ ወይ?	አዎን-----------1<br>አይደለም---------0

ክፍል አራት፡ በባለቤት/በአጋር የሚደርስ ወሲባዊ ጥቃት፡፡ ከዚህ በታች የቀረቡት ጥያቄዎች በሙሉ የሚያመለክቱት ስለ አሁኑ እርግዝና ብቻ ነው፡፡

ተ.ቁ	ጥያቄዎች	መልስ
401ሀ	በዚህ እርግዝና ወቅት ባለቤትዎ በአካል አስገድዶዎት የግብረ ስጋ ግንኙነት አድርገው ያውቃሉ ወይ?	አዎን-----------1<br>አይደለም---------0
401ለ	ከዚህ እርግዝና በፊት በባለፈው አመት በማንኛውም ጊዜ በአሁኑ ባለቤትዎት እንደዚህ አይነት ጥቃት አጋጥሞዎት ያውቃል ወይ?	አዎን-----------1<br>አይደለም---------0
402ሀ	በዚህ እርግዝና ወቅት ባለቤትዎ የሆነ ነገር ሊያደርግ ይችላል ብለው ፈርተው ሳይፈልጉ የግብረ ስጋ ግንኙነት አድርገው ያውቃሉ ወይ?	አዎን-----------1<br>አይደለም---------0
402ለ	ከዚህ እርግዝና በፊት በባለፈው አመት በማንኛውም ጊዜ በአሁኑ ባለቤትዎት እንደዚህ አይነት ጥቃት አጋጥሞዎት ያውቃል ወይ?	አዎን-----------1<br>አይደለም---------0

403ሀ	በዚህ እርግዝና ወቅት በባለቤትዎ ተገደው ወሲባዊ የሆነ ዝቅ የሚያደርግ ወይም የሚያዋርድ ነገር አድርገው ያውቃሉ ወይ?	አዎን------------1<br>አይደለም----------0
403ለ	ከዚህ እርግዝና በፊት በባለፈው አመት በማንኛውም ጊዜ በአሁኑ ባለቤትዎት እንደዚህ አይነት ጥቃት አጋጥሞዎት ያውቃል ወይ?	አዎን------------1<br>አይደለም----------0
404	እናትየው ከላይ ካሉት ጥያቄዎች(ክፍል ሁለት/ክፍል ሶስት/ክፍል አራት) ለአንዱም አዎ ብላ ከመለሰች ቀጥሎ ያለውን ጥያቄ ይጠይቁ ከማርገዝዎ በፊት ከነበረው ጋር ሲወዳደር አሁን በእርግዝናው ጊዜ ጥቃቱ ቀንሷል ወይስ ያው ነው ወይስ ጨምሯል?	ቀንሷል------------1<br>ያው ነው---------2<br>ጨምሯል---------3<br>አላውቅም--------4

ክፍል አምስት፡ የመደበር/የመከፋት ስሜት

ባለፉት ሰባት ቀናት ውስጥ ዛሬን ጨምሮ ይሰማዎት የነበረውን ስሜት ይንገሩን፡፡

ተ.ቁ	ጥያቄዎች	ምላሾች
501	ባለፉት ሰባት ቀናት ውስጥ ለመሳቅና የነገሮችን አስቂኝ ጎን ለማየት ችለው ነበር?<br>ለምሳሌ እንደተለመደው በሚያስቁዎት ነገሮች ላይ መሳቅ ችለው ነበር?	አዎን በተቻለኝ መጠን ሁሌም እንደማደርገው---------------------------------------0<br>አሁን እንኳን ያን ያክል አይደለም--1<br>አይደለም በእርግጥ አሁን ያን ያክል አይደለም---2<br>እንዲያውም --3
502	ባለፉት ሰባት ቀናት ውስጥ ነገሮችን በመደሰት ይመለከቱ ነበር?<br>ለምሳሌ ላምዎት ብታረግዝ የምትወልደበትን በጉጉት ይጠብቃሉ? ሌላ ምሳሌ የገበያ ቀንን በጉጉት ይጠብቃሉ?	አዎን በተቻለኝ መጠን ሁሌም እንደማደርገው ------------------------------------0<br>አደርገው ከነበረው ባነሰ --1<br>አይደለም በእርግጥ አደርገው ከነበረው ባነሰ---2<br>እንዲያውም ---3
503	ባለፉት ሰባት ቀናት ውስጥ ነገሮች ሲበለሻሹ ራስዎን አላግባብ ኮንነው ነበር?<br>ለምሳሌ ልጅዎት ቢታመም	አይደለም በጣም ብዙ ጊዜ አይደለም---0<br>በፍጹም አይደለም--1

	ራስዎን ይኮንናሉ?	አዎን በጣም ብዙ ጊዜ--2<br>አንዳንዴ---3
504	ባለፉት ሰባት ቀናት ውስጥ ያለ በቂ ምክንያት ተጨንቀው ነበር?	አይደለም በፍጹም---0<br>እምብዛም/ላመል--1<br>አዎን አንዳንዴ---2<br>በጣም በተደጋጋሚ--3
505	ባለፉት ሰባት ቀናት ውስጥ ያለ በቂና በጣም ጥሩ ምክንያት ፍርሃት ተሰምቶዎት ነበር?	አይደለም በፍጹም---0<br>ብዙም አይደለም---1<br>አዎን አንዳንዴ ---2<br>በጣም ብዙ ጊዜ---3
506	ባለፉት ሰባት ቀናት ውስጥ ነገሮች ሁሉ ተመሰቃቅለውብዎት ነበር?	አይደለም እንደ ሁልጊዜው እየተወጣሁት ነው -----------------------------------0<br>ብዙ ጊዜ በጥሩ ሁኔታ እወጣለሁ---1<br>አዎን አንዳንዴ እንደ ሁል ጊዜው አልወጣም---2<br>በአብዛኛው እንደ ሁል ጊዜው አልወጣም--3
507	ባለፉት ሰባት ቀናት ውስጥ እንቅልፍ ከማጣትዎት የተነሳ ደስተኛ አልነበሩም?	አይደለም አይደረግም---0<br>በተደጋጋሚ አይደለም ---1<br>አዎን አንዳንዴ--2<br>በአብዛኛው---3
508	ባለፉት ሰባት ቀናት ውስጥ ሀዘን ተሰምቶዎት ነበር?	አይደለም አይደረግም---0<br>በተደጋጋሚ አይደለም --1<br>አዎን አንዳንዴ---2<br>በአብዛኛው--3
509	ባለፉት ሰባት ቀናት ውስጥ በጣም ሀዘን ስለተሰማዎት አለቅሰው ነበር?	አይደለም በፍጹም---0<br>አንዳንድ ጊዜ ብቻ---1

		አዎን በተደጋጋሚ---2<br>በአብዛኛው --3
510	ባለፉት ሰባት ቀናት ውስጥ እራስዎን የመጉዳት ሃሳብ አስበው ነበር?	አይደለም በፍጹም--0<br>በመጠኑ ሁሌም--1<br>አዎን አንዳንዴ --2<br>በተደጋጋሚ---3

ክፍል ስድስት: ከተለያዩ ሰዎች የተገኘ ማህበራዊ ድጋፍ

ከተለያዩ ሰዎች ስለአገኙት ማህበራዊ ድጋፍ ይንገሩን

		ሁልጊዜ	አብዛኛውን ጊዜ	አንዳንድ ጊዜ	አልፎ አልፎ	በጭራሽ
601	የሚረዱዎት መልካም ጓደኞች አሉዎት?	5	4	3	2	1
602	ቤተሰብዎት ሊረዳዎት ሁልጊዜ በቅርብ ይገኛል?	5	4	3	2	1
603	ባለቤትዎት/አጋርዎትበጣም ይረዱዎታል?	5	4	3	2	1
604	በእርስዎ እና በባለቤትዎት/አጋርዎት መካከል አለመግባባት አለ?	1	2	3	4	5
605	ባለቤትዎት/አጋርዎት እንደሚቆጣጠርዎት ይሰማዎታል?	1	2	3	4	5
606	በባለቤትዎት/አጋርዎት እንደሚፈቀሩ ይሰማዎታል?	5	4	3	2	1

ጊዜዎትን ሰውተው ጥያቄዎቹን ለመመለስ ላደረጉት ጥረት እናመሰግናለን፡፡

አበቃ
